# *In vitro* and *in vivo* host range of *Anopheles gambiae* densovirus (AgDNV)

**DOI:** 10.1038/srep12701

**Published:** 2015-07-29

**Authors:** Yasutsugu Suzuki, Tapan K. Barik, Rebecca M. Johnson, Jason L. Rasgon

**Affiliations:** 1 Department of Entomology, Center for Infectious Disease Dynamics and the Huck Institutes of the Life Sciences, Pennsylvania State University, University Park, Pennsylvania, 16802, United States of America; 2 Post Graduate Department of Zoology, Berhampur University, Berhampur, Odisha 760007, India

## Abstract

AgDNV is a powerful gene transduction tool and potential biological control agent for *Anopheles* mosquitoes. Using a GFP reporter virus system, we investigated AgDNV host range specificity in four arthropod cell lines (derived from *An. gambiae*, *Aedes albopictus* and *Drosophila melanogaster*) and six mosquito species from 3 genera (*An. gambiae*, *An. arabiensis*, *An. stephensi*, *Ae. albopictus*, *Ae. aegypti* and *Culex tarsalis*). *In vitro*, efficient viral invasion, replication and GFP expression was only observed in MOS55 *An. gambiae* cells. *In vivo*, high levels of GFP were observed in *An. gambiae* mosquitoes. Intermediate levels of GFP were observed in the closely related species *An. arabiensis*. Low levels of GFP were observed in *An. stephensi*, *Ae. albopictus*, *Ae. aegypti* and *Cx. tarsalis.* These results suggest that AgDNV is a specific gene transduction tool for members of the *An. gambiae* species complex, and could be potentially developed into a biocontrol agent with minimal off-target effects.

Densoviruses (DNVs) are non-enveloped single-stranded DNA viruses in the family *Parvoviridae.* DNVs are broadly distributed in invertebrates and are often pathogenic to their hosts^1^,^2^,^3^,^4^,^5^,^6^. Many DNVs have been isolated from various laboratory and field mosquitoes and cell lines^1^,^7^,^8^,^9^. The *Anopheles gambiae* densovirus (AgDNV) is highly infectious and capable of transducing exogenous genes in *An. gambiae*, the major human malaria vector in Sub-Saharan Africa^9^,^10^. Unlike most mosquito densoviruses, AgDNV exhibits negligible pathology in *An. gambiae*^11^. These features make AgDNV an attractive candidate for paratransgenesis, an approach that renders insects refractory to pathogens by using transgenic microbes^12^,^13^,^14^. The use of paratransgenesis in the field needs to be considered carefully and unwanted side effects such as off-target infections need to be investigated. Understanding basic aspects of viral ecology such as host range is crucial to evaluate feasibility of viral paratransgenesis in the field.

Densovirus host range has been studied with the *Aedes aegypti* densovirus (AeDNV) and *Aedes albopictus* densovirus (AalDNV). AeDNV and AalDNV are infectious to *Aedes*, *Culex* and *Culiseta* mosquitoes, but are not infectious to other insects or vertebrates^1^,^6^. Among mosquitoes, *Ae. aegypti* and *Ae. albopictus* show relatively high susceptibility to multiple mosquito densoviruses^5^,^15^,^16^,^17^. Ward *et al.* used a recombinant AeDNV expressing green fluorescent protein (GFP), and demonstrated that AeDNV can fully disseminate in *Ae. aegypti* but only infects the anal papillae or bristle cells and does not disseminate in *An. gambiae*^18^. Previous work by our lab led to the isolation of AgDNV and the development of GFP-expressing recombinant virus, which is capable of efficiently infecting and disseminating in *An. gambiae*^9^,^10^. In this study, we used this system to investigate the host range of AgDNV in multiple invertebrate cell lines and mosquito species.

## Results

### *In vitro* AgDNV host specificity

**Figure 1:**
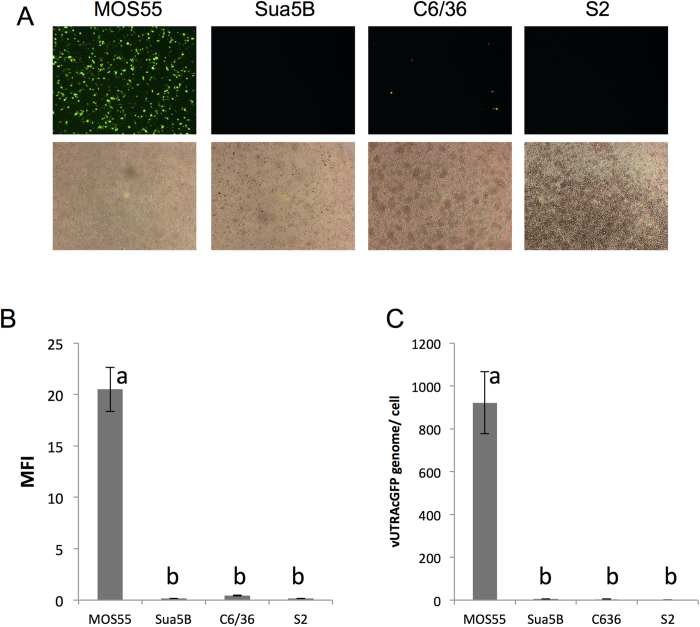
Infection of insect cell lines with vUTRAcGFP. GFP expression was (**A**) visualized by fluorescent microscopy and (**B**) quantified by flow cytometry analysis in MOS55, Sua5B, C6/36 and S2 cells. MFI = mean fluorescence intensity. (**C**) vUTRAcGFP viral DNA copy number was quantified by qPCR analysis. Graphs show data mean and standard deviations. Data were analyzed by Analysis of Variance (ANOVA) with Bonferroni’s correction for multiple comparisons. Letters represent statistical significance (P < 0.05).

MOS55 (*An. gambiae*), Sua5B (*An. gambiae*), C6/36 (*Ae. albopictus*) and S2 (*Drosophila melanogaster*) cell lines were infected with 1 × 10^9^ virions of recombinant GFP-expressing AgDNV (vUTRAcGFP)^10^. Three days post-infection, GFP expression levels were examined using fluorescence microscopy, flow cytometry and quantitative PCR (qPCR). MOS55 cells showed the highest GFP fluorescence (Fig. 1A,B). Low levels of GFP were observed in C6/36 cells and no fluorescence was observed in Sua5B or S2 cells (Fig. 1A,B). Using qPCR, levels of viral DNA copies of vUTRAcGFP matched results obtained by flow cytometry (Fig. 1C).

### *In vivo* AgDNV host specificity

**Figure 2:**
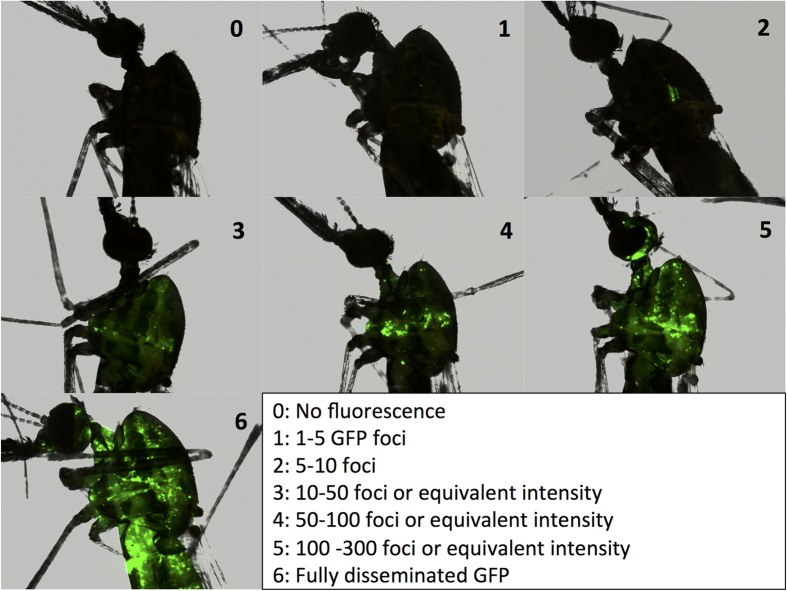
Representative images of vUTRAcGFP-infected mosquitoes for scoring GFP expression. Fluorescence levels were categorized into 7 categories (0–6) based on the indicated criteria. *An. stephensi* (scores, 0–2) and *An. gambiae* (scores, 3–6) are shown as representative examples.

**Figure 3:**
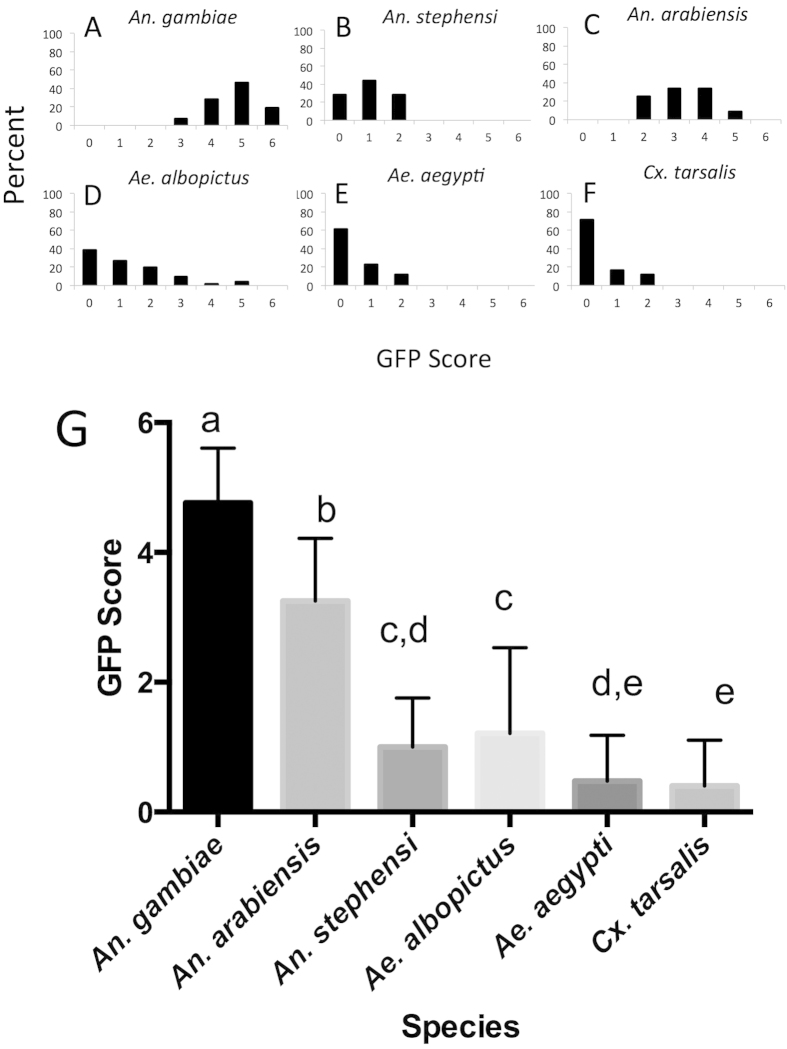
Comparison of GFP infection scores in six mosquito species. (**A**) *An. gambiae*, (**B**) *An. stephensi*, (**C**) *An. arabiensis*, (**D**) *Ae. albopictus*, (**E**) *Ae. aegypti* and (**F**) *Cx. tarsalis*. (**G**) Mean infection score for each mosquito species. Data were analyzed by ANOVA with Bonferroni’s correction for multiple comparisons. Letters represent statistical significance (P < 0.05).

We next investigated viral host range among mosquito species *in vivo* using *An. gambiae, An. stephensi, An. arabiensis*, *Ae. aegypti, Ae. albopictus* and *Culex tarsalis*. 40–50 adult mosquitoes of each species were injected with 1 × 10^7^ of vUTRAcGFP. At 7 days post injection, mosquitoes were visually examined for GFP expression using fluorescence microscopy. We defined a seven category scoring criteria (0–6) for the level of fluorescence expression in individual mosquitoes (Fig. 2). This scoring system allowed us to compare the viral infection levels semi-quantitatively and analyze the distribution of GFP expression level within each species. The known permissive mosquito, *An. gambiae,* exhibited scores ranging from 3 to 6 with an average score of 4.8 (Fig. 3A,G). The closely related species *An. arabiensis* exhibited scores ranging from 2 to 5 with an average score of 3.3 (Fig. 3C,G). In other mosquito species, the distributions were shifted and had statistically significantly lower ranges (Fig. 3). *An. stephensi*, the closest relative of *An. gambiae* and *An. arabiensis* examined in this study, had an average score of only 1.0 (Fig. 3B,G). *Ae. aegypti*, *Ae. albopictus* and *Cx. tarsalis* had average scores of 0.47, 1.2 and 0.40 respectively (Fig. 3D–G).

## Discussion

Although AgDNV was originally isolated from *An. gambiae* Sua5B cells^9^, no fluorescence or viral DNA was detected after vUTRAcGFP infection of this cell line. However, Sua5B cells are permissive to viral replication of the naturally occurring virus present in the cell line or if transfected with a recombinant infectious clone plasmid^9^. These results suggest that Sua5B cells may have been originally infected with AgDNV from the original mosquito colony from which the cell line was established, however, during development of the cell line and/or over long-term serial passage the cells lost essential host factors (such as receptors) required for new infection. In contrast, the *An. gambiae* MOS55 cell line retains these factors and is permissive to infection. Comparison of these two cell lines may help identify the specific receptors required for AgDNV entry into cells.

The *An. gambiae* species complex consists of at least seven morphologically identical mosquito species, to which both *An. gambiae* and *An. arabiensis* belong^19^. We had initially hypothesized that AgDNV would in general infect Anopheline mosquitoes better than species belonging to other genera. However, this was not the case. While *An. gambiae*, and to a lesser extent *An. arabiensis* were susceptible to infection, the congeneric species *An. stephensi* was refractory to infection (Fig. 3B,G). *An. stephensi* is the major Asian vector of human malaria and is not part of the gambiae complex^19^. These observations suggest that AgDNV is specifically adapted to infect *An. gambiae* and closely related species.

We unexpectedly observed intermediate to high levels of GFP expression (scores of 3 to 5) in a low percentage of *Ae. albopictus* individuals (Fig. 3D), leading to a significantly higher mean infection score for *Ae. albopictus* compared to *Ae. aegypti* or *Cx. tarsalis* (P < 0.05) (Fig. 3D–G). These results complement our cell line data, where C6/36 cells (derived from *Ae. albopictus*) were also minimally permissive to viral infection. To date, there are no reports detailing the molecular mechanisms underlying host specificity of mosquito densoviruses. Clathrin-mediated endocytosis has been shown to be important for infection for mammalian and insect parvoviruses such as canine parvovirus (CPV) and *Junonia coenia* densovirus (JcDNV)^20^,^21^,^22^. The clathrin-mediated endocytosis pathway is likely used by mosquito densoviruses as well. Structural variation of the receptors and downstream molecules could determine the host specificity of AgDNV among mosquito species and remains to be investigated.

Unlike other densoviruses that are highly pathogenic to their hosts, AgDNV has a negligible impact on the life span of *An. gambiae*^1^,^2^,^3^,^4^,^5^,^6^,^11^. The highly infectious but non-pathogenic and specific nature of the interaction between *An. gambiae* and AgDNV suggests a history of co-evolution and host-specific adaptation in this system that is distinct from other studied mosquito densoviruses. Further experiments to elucidate the molecular mechanisms of this observed specificity will provide mechanistic insights into the evolution of host-specific pathogens, and will inform on the utility of using DNVs for targeted biocontrol of vector mosquitoes in the field.

## Methods

### Transducing virus production

Virions of recombinant GFP-expressing AgDNV (vUTRAcGFP) were produced by co-transfection of MOS55 cells with the recombinant virus plasmid pUTRAcGFP and the wild type AgDNV helper plasmid pBAgα as described^9^,^10^. Viral titer for these infection experiments was determined with qPCR using a standard curve as previously described^10^. Briefly, DNV samples were TURBO DNase (Ambion) treated to digest plasmid DNAs. Total DNA was extracted using DNEasy kits (Qiagen). qPCR was performed using the Quantitect SYBR Green Kit (Qiagen) on a Rotor-Gene Q (Qiagen) with EGFP primers: 5′ TCA-AGA-TCC-GCC-ACA-ACA-TC 3′, 5′ TTC-TCG-TTG-GGG-TCT-TTG-CT 3′. A standard curve was created using a dilution series of pUTRAcGFP ranging from 10^3^ to 10^8^ copies.

### *In vitro* AgDNV infection quantitation

MOS55 (*An. gambiae*), Sua5B (*An. gambiae*), C6/36 (*Ae. albopictus*) and S2 (*Drosophila melanogaster*) cell lines were cultured in Schneider’s media with 10% fetal bovine serum. Cells were infected with 1 × 10^9^ virions of recombinant GFP-expressing AgDNV (vUTRAcGFP)^10^. Viral DNA level in infected cells was determined by qPCR as described above. GFP mean fluorescence intensity (MFI) was determined using flow cytometry with FlowJo software. Statistical differences between treatments were determined using analysis of variance (ANOVA) with Bonferroni’s correction for multiple comparisons.

### *In vivo* AgDNV infection quantitation

Mosquitoes of each species (*An. gambiae* [Keele strain]*, An. stephensi* [Liston strain]*, An. arabiensis* [Dongola strain], *Ae. aegypti* [Rock strain]*, Ae. albopictus* [Houston strain] and *Culex tarsalis* [Yolo strain]) were held at 27 **°**C and 80% relative humidity and were maintained on expired human blood or commercially obtained bovine blood using a membrane feeding system, and were allowed access to 10% sucrose solution *ad libitum* through a cotton wick. For each species, 3–5 day old females were anesthetized by chilling and injected with 1 × 10^7^ vUTRAcGFP using a glass capillary needle. Injected mosquitoes were held at 27 °C and 80% relative humidity with 10% sucrose. A 7 category scoring scale (Fig. 2) was used to visually quantify GFP expression using an Olympus BX40 epifluorescent microscope at 7 days post-injection. Statistical differences between treatments were determined using analysis of variance (ANOVA) with Bonferroni’s correction for multiple comparisons. *An. gambiae* injected with media were used as a negative control.

## Additional Information

**How to cite this article**: Suzuki, Y. *et al.* *In vitro* and *in vivo* host range of *Anopheles gambiae* densovirus (AgDNV). *Sci. Rep.* **5**, 12701; doi: 10.1038/srep12701 (2015).
